# Compact Snapshot Hyperspectral Imaging With Neural Dispersion‐Engineered Metalens

**DOI:** 10.1002/nap2.70245

**Published:** 2026-08-07

**Authors:** Peng Liu, Jiaru Chu, Yuhang Chen

**Affiliations:** ^1^ Department of Precision Machinery and Precision Instrumentation University of Science and Technology of China Hefei China

**Keywords:** computational spectral imaging, dispersion engineering, end‐to‐end joint optimization, metalens

## Abstract

There is a growing demand for ultra‐compact, low‐cost, and high‐fidelity snapshot hyperspectral imaging devices across various fields. However, conventional systems struggle to fulfill all these evolving requirements. Although a single diffractive optical element offers a compact solution, its degrees of freedom in light phase modulation are limited. Here, we exploit wavelength‐dependent characteristics of the point spread function (PSF) to design a dispersion‐engineered metalens that serves as a key element for hyperspectral imaging. The PSF of the metalens is tailored to undergo an extra lateral shift as a function of wavelength, thereby encoding richer spectral information. An efficient differentiable computational model is developed to simulate the hyperspectral imaging process, together with a downstream spectral reconstruction network. The metalens structure and the reconstruction network are subsequently optimized in an end‐to‐end deep learning joint optimization framework. We fabricated the optimal metalens using two‐photon grayscale lithography and built a prototype hyperspectral imaging camera. Both indoor and outdoor experimental results validated its outstanding spatial‐spectral reconstruction performance, highlighting the effectiveness in practical applications.

## Graphical abstract

A dispersion‐engineered metalens, designed via an end‐to‐end deep learning framework, encodes spectral information through wavelength‐dependent PSF shifts. Fabricated by two‐photon grayscale lithography, the ultrathin device demonstrates outstanding spatial‐spectral reconstruction in both indoor and outdoor scenes, positioning it as a promising candidate for compact, high‐fidelity snapshot hyperspectral imaging.

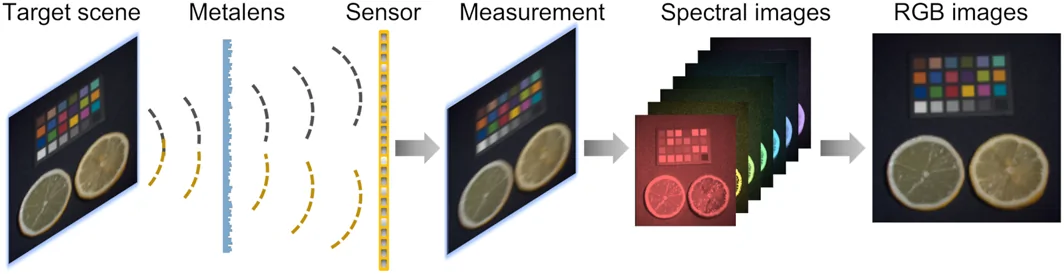

## 1 Introduction

Hyperspectral imaging is an advanced optical imaging technology, which captures not only spatial details but also continuous spectral data at each pixel, forming a three‐dimensional (3D) data cube that weaves together spatial and spectral information [1, 2, 3]. Driven by its superior information acquisition capability over visible color (RGB) imaging, hyperspectral imaging has been widely applied in fields such as remote sensing, agricultural monitoring, biomedical imaging, food safety, environmental surveillance, defense, and security [4, 5, 6, 7]. However, conventional hyperspectral imaging generally relies on a set of dispersion optics, relay lenses, spectral filters, and scanning devices with rather complex optical setups, resulting in bulky equipment and high cost. Furthermore, the imaging speed is often constrained due to the scanning mechanism, which is inefficient and thus prohibitive for real‐time imaging of dynamic scenes [8].

To meet the growing demand for real‐time spectral data acquisition, snapshot hyperspectral imaging has emerged as a promising solution. This approach overcomes the inherent speed limitation of traditional scanning hyperspectral imaging systems by capturing both spectral and spatial information of a scene in a single shot. Representative implementations include the coded aperture snapshot spectral imager, the prism‐mask imaging spectrometer, and the Fourier transform snapshot spectral imager, among others [9, 10, 11]. It is worth noting that standard refractive and reflective optical elements continue to form the foundational components of these systems, which often give rise to bulky configurations and large form factors. Consequently, they are challenged by the stringent size and integration constraints of consumer‐grade applications.

Achieving compact snapshot spectral imaging systems entails employing a single optical element to accomplish the modulation and encoding of spectral information. Such approaches fall primarily into two categories: filter‐based encoding and dispersion‐based encoding. One common filter‐based implementation directly integrates a spectral filter array onto the image sensor to reconstruct the spectra. This array can be regarded as an extended form of the Bayer filter used in RGB imaging, containing multiple narrowband or broadband spectral filters [12, 13]. However, as the number of filters increases for accurate spectral reconstruction, the spatial resolution of reconstructed hyperspectral images decreases significantly. As for dispersion‐based encoding, it captures the spectral information by manipulating the wavelength‐dependent dispersion of the point spread function (PSF). Recent advancements have utilized diffractive optical elements (DOEs) with wavelength‐dependent PSFs to encode spectral information [14, 15, 16]. However, constrained by their micron‐scale feature sizes, DOEs exhibit limited flexibility in phase modulation and therefore offer only restricted capability for encoding spectral information. Specifically, current DOE‐based PSF dispersion modulation is confined to the radial direction because imparting additional phase profiles may cause overly steep phase gradients, which in turn induce higher‐order diffraction and reduce optical efficiency. In contrast, the emerging planar optical metasurfaces enable more flexible optical field manipulation, offering a highly compelling alternative for ultra‐compact hyperspectral imaging [17].

Metasurfaces, comprised of subwavelength meta‐unit arrays, enable tailored control over light's amplitude, phase, and polarization at the nanoscale. Compared to other refractive and diffractive optical elements, metasurfaces offer greater precision and flexibility in light modulation while being more compact and lightweight [18, 19]. All these distinctive advantages make them highly attractive for a wide range of applications, spanning diverse fields from 3D/polarization imaging to holographic display and from achromatic imaging to spectroscopy [20, 21, 22, 23, 24]. In spectral imaging, however, they have yet to mature for consumer‐level implementations, where the achieved image quality also remains underdeveloped. Furthermore, many existing works treat the metasurface designs and the spectral reconstruction algorithms as separate processes. This disconnect renders it challenging to fully leverage the metasurface dispersion information at the design stage for accurate spectral image reconstruction [25, 26, 27, 28]. Currently, some cutting‐edge studies aim to be task‐driven by jointly modeling optical design and algorithmic processing as a unified framework, enabling end‐to‐end optimization to build a system with optimal performance [29, 30]. In terms of metasurface fabrication, the mainstream approach is based on electron beam lithography and etching, although a few studies have explored the use of femtosecond laser direct‐write lithography to enable low‐cost and rapid prototyping [31, 32].

In this work, we propose a dispersion‐engineered metalens, a functional metasurface subset, for hyperspectral imaging. In contrast to most existing approaches that rely on manually designed metalens structures and hyperspectral reconstruction algorithms, we develop a differentiable spectral imaging model and employ an end‐to‐end joint learning framework to co‐optimize both the metalens structure and the reconstruction algorithm. In addition, rather than using metasurfaces as an array of narrowband or uncorrelated broadband spectral filters, which may degrade spatial resolution, we use the phase‐encoding approach [33, 34, 35]. In this design, the metalens serves as both an imaging component and a spectral encoder by leveraging the wavelength‐dependent lateral shift of its PSF. Therefore, the optical system is simplified, whereas the spatial resolution of the image sensor is maximally preserved.

Comparative simulations verified that the proposed method outperforms traditional separated designs and existing end‐to‐end joint optimization methods without the special metalens dispersion engineering. We further fabricated the learned metalens using two‐photon grayscale lithography and developed a prototype hyperspectral camera to validate the feasibility. Hyperspectral imaging and reconstruction experiments performed in both indoor and outdoor environments highlight its robustness and potential for practical usages. We believe that the proposed method provides an innovative and efficient solution for compact snapshot hyperspectral imaging, significantly advancing its deployments across a broader range of fields.

Specifically, we make the following contributions: (i) We propose a snapshot hyperspectral imaging system based on a dispersion‐engineered metalens, which precisely modulates the PSF dispersion along both the radial and transverse directions, thereby encoding richer spectral information. (ii) We construct a fully differentiable hyperspectral imaging framework that incorporates an efficient imaging model together with a downstream reconstruction network. Within this framework, the metalens structure and the spectral reconstruction network are jointly optimized in an end‐to‐end manner to achieve optimal spectral encoding and reconstruction performance. (iii) We evaluate the proposed method and compare it with existing diffractive‐lens‐based encoding implementations. The results show that, for the reconstruction task involving 25 spectral channels, the peak signal‐to‐noise ratio improves by approximately 4 dB. (iv) We build a prototype system and conduct comparative experiments against a commercial spectrometer. Both indoor and outdoor experiments validate the effectiveness and accuracy of practical hyperspectral imaging.

## 2 Results

### 2.1 Dispersion‐Engineered Hyperspectral Imaging Framework

The proposed snapshot hyperspectral imaging system consists of two main parts including the optical hardware and the reconstruction network, with the overall process illustrated in Figure 1. In the front‐end optical part, a dispersion‐engineered metalens is designed with distinct PSFs across different wavelength bands, accompanied by wavelength‐dependent lateral PSF displacements, enabling effective encoding. The spectral information of the scene is first encoded by the metalens and then projected onto the CMOS sensor of the RGB camera, yielding a raw measurement. This raw image is routed to a downstream hyperspectral reconstruction network, which processes and decodes it to recover the full spectral content of the scene as well as the clean color image.

**FIGURE 1:**
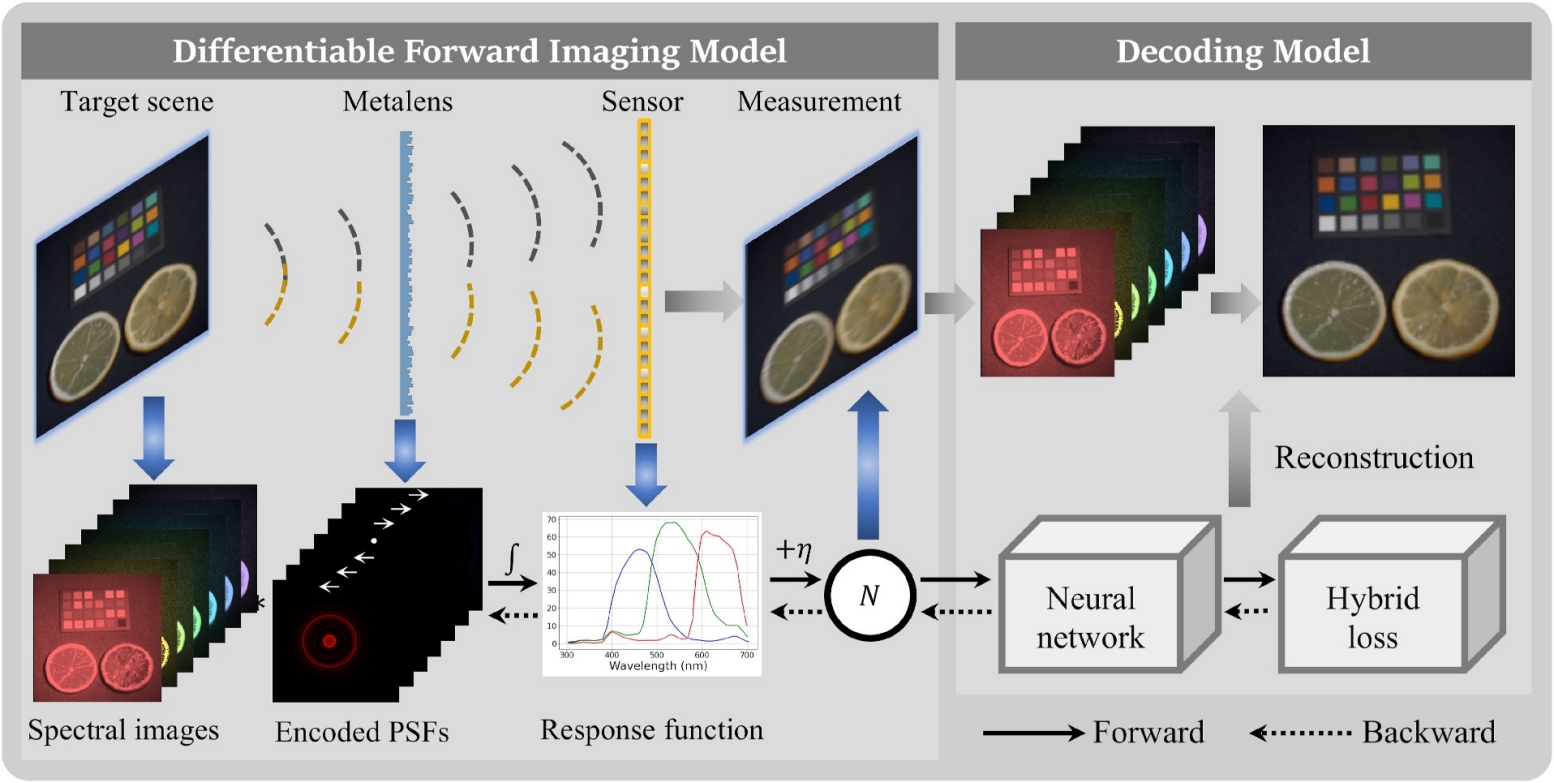
Hyperspectral imaging framework based on the dispersion‐engineered metalens.

In simulating the imaging process, the forward spectral encoding is implemented by convolving the PSFs at different wavelengths with their corresponding ground‐truth hyperspectral images. Subsequently, the convolution results across all spectral bands are weighted and integrated according to the spectral response curves of the CMOS sensor to generate a noise‐free image. Random noise is then added to simulate realistic imaging conditions. In the downstream stage, a multi‐resolution convolutional neural network is designed to decode the sensor image for spectral reconstruction, followed by the synthesis of the sharp RGB image. By introducing a hybrid loss function, the entire framework enables end‐to‐end joint optimization of the metalens structure and the downstream neural network for accurate snapshot hyperspectral imaging.

### 2.2 Differentiable Forward Imaging Model

In this section, we establish a differentiable forward spectral imaging model based on the dispersion‐engineered metalens. First, starting from a source point in the scene, the optical field at a plane (*u*, *v*) with the propagation distance *z* from the point source can be expressed as follows:

(1) $$U_{\text{obj}}\left(u,v\right)=Ae^{\text{ikz}}e^{i\frac{k}{2z}\left(u^{2}+v^{2}\right)},$$

where *A* is the amplitude, *k* is the wave number, and *i* is the imaginary unit.

As the optical field propagates through the metalens, its phase profile undergoes a corresponding modulation, where the imparted phase can be decomposed into a fundamental focusing phase $\varphi_{\text{focus}}$ and a linear gradient phase $\varphi_{\text{shift}}$. The fundamental focusing phase enables basic imaging and partial spectral encoding and is optimized within the end‐to‐end framework, whereas the linear gradient phase introduces a lateral, wavelength‐dependent dispersion to further enrich the spectral encoding information. The phase modulation function of the metalens is then described by

(2) $$\varphi_{\text{total}}=\varphi_{\text{focus}}+\varphi_{\text{shift}},$$

(3) $$\varphi_{\text{shift}}=\frac{2\pi }{\lambda s}\Delta_{\text{shift}}u,$$

where $\Delta_{\text{shift}}$ denotes the lateral displacement of the PSF at the assigned central wavelength in the spectral range. The modulated phase delay $\varphi_{\text{total}}$ of the metalens is governed by the height profile h(u,v) of the array of meta‐units as $\varphi_{\text{total}}\left(u,v,\lambda \right)=k\Delta n\left(\lambda \right)h\left(u,v\right)$, where Δn(λ) denotes the refractive index contrast of the metalens material with respect to air. Certainly, other geometric parameters of the meta‐units can be tuned to achieve the same phase modulation while maintaining a fixed height. However, given the convenient fabrication by two‐photon grayscale lithography, we opt to vary the meta‐unit height. The PSFs at different wavelengths exhibit distinct lateral displacements, thereby encoding more spectral information of the scene across different bands. Following modulation with the metalens, the optical field can be obtained as follows:

(4) $$U^{\prime }\left(u,v\right)=U_{\text{obj}}\left(u,v\right)p\left(u,v\right)e^{i\varphi_{\text{total}}},$$

where p(u,v) denotes the aperture function $\text{circ}\left(\sqrt{u^{2}+v^{2}}/R\right)$ with a radius of *R*. After Fresnel diffraction propagation to the sensor plane with a distance of *s* from the metalens, the optical field is

(5) $$U_{\text{sensor}}\left(x,y,\lambda \right)=\frac{e^{iks}}{i\lambda s}\iint U^{\prime }\left(u,v\right)e^{i\frac{k}{2s}\left[{\left(x-u\right)}^{2}+{\left(y-v\right)}^{2})\right]}\mathrm{dudv},$$

Expanding the above equation and removing the constant terms yield

(6) $$U_{\text{sensor}}\left(x,y,\lambda \right)\propto \iint U^{\prime }\left(u,v\right)e^{i\frac{k}{2s}\left(u^{2}+v^{2}\right)}e^{-i\frac{k}{s}\left(ux+vy\right)}\mathrm{dudv}.$$

Then, the PSF of the imaging system can be computed by

(7) $$\text{PSF}\left(x,y,\lambda \right)\propto {\left|U_{\text{sensor}}\left(x,y,\lambda \right)\right|}^{2}.$$

It is worth noting that conventional algorithms often face significant challenges in computing this PSF. On one hand, the metalens contains on the order of 120 million individual meta‐units, making full‐scale optimization extremely difficult and computationally demanding. On the other hand, the introduction of the linear gradient phase imposes higher sampling requirements during simulations, further increasing the computational and optimization burden. Therefore, we implemented the following strategies. First, by imposing a rotational symmetry constraint on the metalens, the number of design parameters was significantly reduced. Second, based on the Fourier shift theorem, the linear gradient phase was directly converted into a displacement of the PSF at the sensor plane. The combination of the two strategies leads to a more efficient way for computing the PSF of the dispersion‐engineered metalens. Detailed derivations are provided in Supporting Information S1: Note S1. Once the PSF is determined, the image captured by the sensor can be simulated.

(8) $$I_{c\in \left\{R,G,B\right\}}=\int_{\lambda_{\min}}^{\lambda_{\max}}{\text{PSF}}_{\lambda }⨂I_{\lambda }\;R_{c}\left(\lambda \right)d\lambda +\eta ,$$

where $I_{\lambda }$ is the hyperspectral image at the wavelength channel λ bounded between $\lambda_{\min}$ and $\lambda_{\max}$, ⨂ is the convolution operator, $R_{c}\left(\lambda \right)$ denotes the response function of the sensor for the red (*R*), green (*G*), and blue (*B*) channels, and η represents the sensor noise.

### 2.3 Hyperspectral Image Reconstruction Network

The spectral information of the scene is encoded into an RGB image by the dispersion‐engineered metalens, and, subsequently, a reconstruction network is established to recover the hyperspectral images. Inspired by the multi‐scale deblurring network [36], we designed a multi‐scale U‐Net to perform the hyperspectral reconstruction task, as illustrated in Supporting Information S1: Figure S1. During the encoding–decoding process, upsampling and downsampling operations are employed to facilitate information exchange across multiple resolution levels. The encoder processes multi‐scale inputs in parallel and produces matching outputs at each scale. The first branch receives the original input, whereas the second and third branches take inputs that are downsampled by factors of two and four, respectively. In the final stage, the three output heads yield hyperspectral images at different scales, which are then fused to reconstruct the full‐resolution hyperspectral images and the RGB image. More detailed information regarding the neural network can be found in Supporting Information S1: Note S2.

### 2.4 Hybrid Loss and Regularization

To train the aforementioned network architecture, we construct a hybrid loss function.

(9) $$L_{\text{total}}=L_{h}+R_{\text{RGB}}+R_{\text{PSF}}+R_{\text{grad}}.$$

The loss function is primarily composed of four components, including the hyperspectral reconstruction loss term $L_{h}$, the RGB regularization term $R_{\text{RGB}}$, the PSF regularization term $R_{\text{PSF}}$, and the gradient regularization term $R_{\text{grad}}$. The first two terms ensure the fidelity of the reconstructed outputs, whereas the third term enforces the feasibility of the PSF design and the final one incorporates a gradient sparsity prior.

The hyperspectral reconstruction loss is formulated based on the spectral angle mapper loss.

(10) $$L_{h}=\frac{1}{N}\sum_{\lambda =1}^{N}\arccos\left(\frac{\langle \hat{I_{\lambda }},I_{\lambda }\rangle }{{\left\|\hat{I_{\lambda }}\right\|}_{2}{\left\|I_{\lambda }\right\|}_{2}}\right),$$

where $I_{\lambda }$ and $\hat{I_{\lambda }}$ are the ground‐truth and the reconstructed hyperspectral images at the wavelength channel λ.

The reconstructed RGB image is synthesized from the estimated hyperspectral images. An RGB regularization term is introduced to constrain the consistency of the reconstructed results in the color space, thereby further improving the accuracy of hyperspectral reconstruction. The RGB regularization term can be expressed as

(11) $$R_{\text{RGB}}=\omega_{1}L_{l1}^{\text{RGB}}+\omega_{2}L_{p}^{\text{RGB}},$$

where $L_{l1}^{\text{RGB}}$ denotes the pixel‐wise $l_{1}$ loss, whereas $L_{p}^{\text{RGB}}$ represents a perceptual loss computed based on a pre‐trained VGG19 network [37]. $\omega_{1}$ and $\omega_{2}$ are the corresponding weights.

To encourage the tight optical field concentration of PSF across different spectral bands and prevent energy leakage during the deep learning optimization, a regularization term is introduced to constrain the PSF magnitude. Note that the PSF in regularization specifically refers to the one without lateral shift. This regularization term can be formulated as

(12) $$R_{\text{PSF}}={\left\|\text{PSF}-{\text{PSF}}_{\text{mask}}\right\|}_{2}^{2},$$

where ${\text{PSF}}_{\text{mask}}$ is the PSF central region with a specified size, extracted using a binary mask.

Given that lateral PSF shifts are introduced to different wavelength bands during the spectral encoding stage, a spectral‐domain spatial gradient regularization term is consequently applied in the reconstruction stage to encourage spatial alignment among the reconstructed hyperspectral images across all bands. It can be expressed as

(13) $$R_{\text{grad}}={\left\|\nabla_{\lambda }\left(\nabla_{xy}\hat{I_{\lambda }}\right)\right\|}_{2}^{2}.$$

### 2.5 Synthetic Assessment

Before conducting practical experiments, we first compared the proposed method with some related approaches on the same hyperspectral image datasets to validate its effectiveness and superiority. In addition, we evaluated the impact of the wavelength‐dependent PSF lateral shift on the final spectral reconstruction performance. To this end, we combined four publicly released datasets for simulation experiments, namely the ICVL, HSDB, CAVE, and KAUST datasets [38, 39, 40, 41]. The merged dataset was randomly split into three subsets, with 576 samples allocated for training, 72 for validation, and 72 for testing. Furthermore, the images were randomly cropped into 512 × 512 patches and augmented using random flips.

To rigorously assess the performance of our approach, we compared it with three representative baselines, including (i) a snapshot hyperspectral imaging method employing a nonoptimized Fresnel lens (NOFL) that does not incorporate special dispersion engineering, (ii) a diffraction‐based snapshot hyperspectral imaging method using end‐to‐end learned quantization‐aware diffractive optics (QDO) [14], and (iii) a snapshot hyperspectral imaging method based on diffractive rotation (DR) [15]. Specifically, the QDO method incorporates height quantization constraints within an end‐to‐end optimization framework to ensure compatibility with fabrication. The DR method employs a pre‐designed DOE whose PSF rotates with wavelength. This wavelength‐dependent PSF rotation provides a key cue for spectral reconstruction [28]. However, the DOE structure is not jointly optimized with the downstream reconstruction network.

For fairness, all methods were trained on the same merged dataset with an identical number of epochs and under the same simulated sensor noise conditions. The QDO method employed the network from its original work, whereas the other two baseline methods and the proposed method utilized the multi‐resolution hyperspectral reconstruction network designed in this work. The inputs for all four methods consisted of spectral data with dimensions of 25 × 512 × 512, which were simulated into the encoded RGB images according to their respective spectral coding schemes, and then decoded by the neural network. Each method reconstructed 25 spectral bands spanning from 420 to 660 nm for demonstration, and the reconstruction quality was quantitatively evaluated using metrics structural similarity index measure (SSIM) and peak signal‐to‐noise ratio (PSNR).

Figure 2 illustrates two representative scenes selected from the CAVE dataset. It is apparent that the RGB images encoded by the three baseline methods are overall blurry, whereas the encoded images from the proposed method exhibit pronounced spectral dispersion at image texture edges, containing richer dispersed spectral information. As observed from the comparison of spectral profiles at the randomly selected pixels, the reconstructed spectra obtained by the proposed method show the closest agreement with the ground‐truth spectra, demonstrating a higher degree of spectral fidelity. In addition, we quantitatively evaluated the spectral reconstruction results of the four methods using SSIM and PSNR metrics, as summarized in Table 1. The proposed approach achieves superior hyperspectral reconstruction quality, with an SSIM of 0.96 and a PSNR of 32.54 dB, confirming a substantial improvement over all the baseline methods. These results underscore its robustness in the joint reconstruction of optical spectra and color images from complex scenes.

**FIGURE 2:**
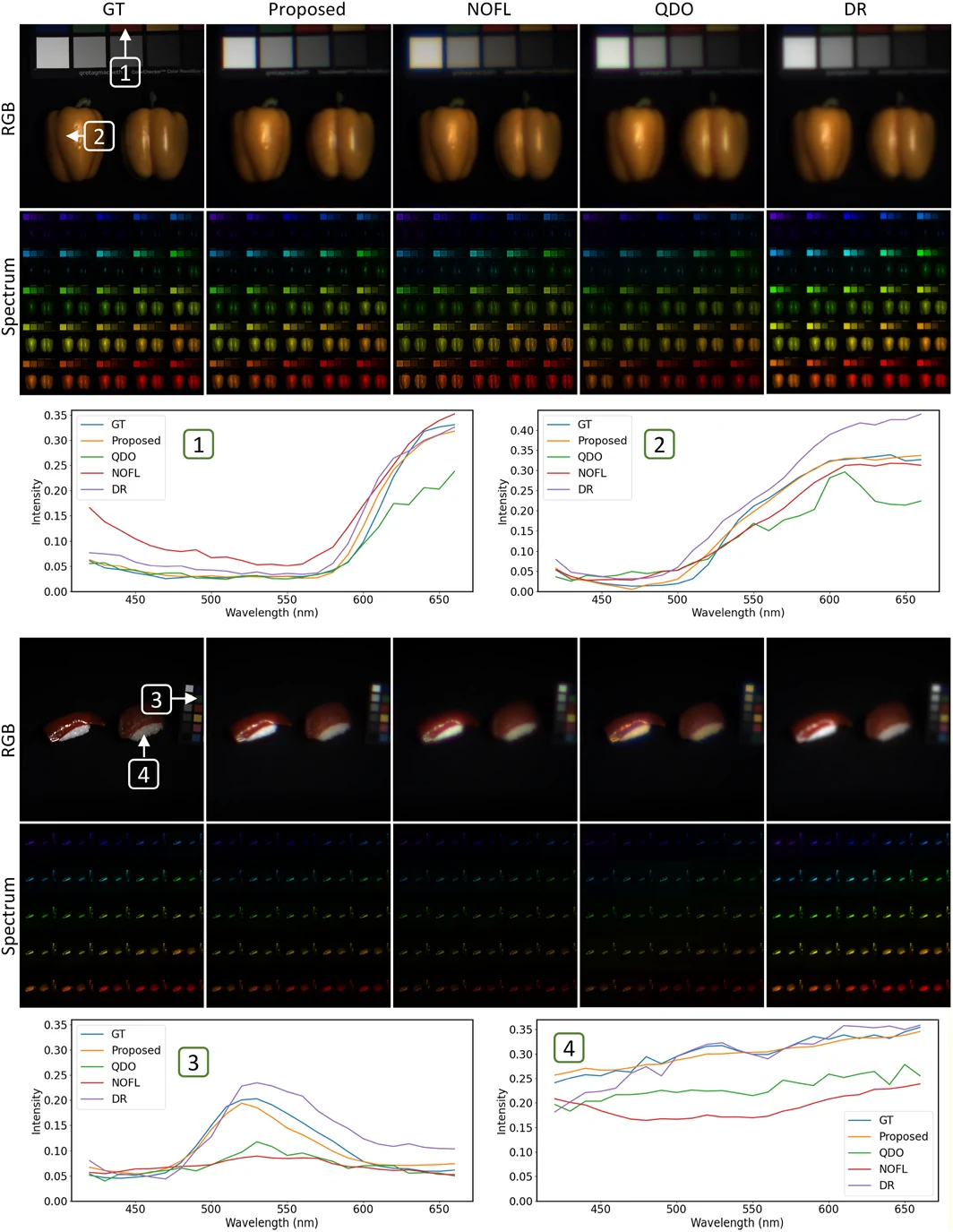
Simulation results on the merged dataset. The first and fourth rows show the ground‐truth and the corresponding spectrally encoded RGB images. The second and fifth rows present the ground‐truth hyperspectral images and the reconstructed results of each method. The third and sixth rows compare the spectral profiles of selected representative pixels across different methods.

**TABLE 1:** Comparison of hyperspectral reconstruction results with other representative methods.

	SSIM	PSNR (dB)
Proposed	0.96	32.54
NOFL	0.83	26.34
QDO	0.88	28.58
DR	0.90	29.06

Subsequently, to further validate the improvement in spectral reconstruction enabled by the dispersion‐engineered metalens, we assessed the performance under different levels of PSF dispersion. Specifically, we varied the lateral displacement $\Delta_{\text{shift}}$ of the PSF at the assigned center wavelength of 546 nm from 0 to 4 mm with an increment of 1 mm while keeping the training dataset and simulated sensor noise identical. The final structural parameters of the metalens were obtained through the established end‐to‐end optimization framework. One typical learned metalens height map with $\Delta_{\text{shift}}$ of 1 mm is presented in Supporting Information S1: Figure S2.

Figure 3 and Table 2 present the qualitative and quantitative comparisons of the hyperspectral reconstruction results under different lateral PSF shifts, respectively. The results demonstrate that applying moderate lateral shifts to the PSF improves hyperspectral reconstruction. However, as the lateral shift further increases, the dispersion becomes more pronounced, resulting in a decline in the reconstruction performance. This indicates that moderate PSF dispersion shift is beneficial for accurate hyperspectral reconstruction, whereas excessive dispersion poses greater challenges to the spectral reconstruction network, causing a degradation in its performance. Given the above observation, we set the optimal lateral PSF displacement at the center wavelength to 1 mm in our final metalens design for practical fabrication.

**FIGURE 3:**
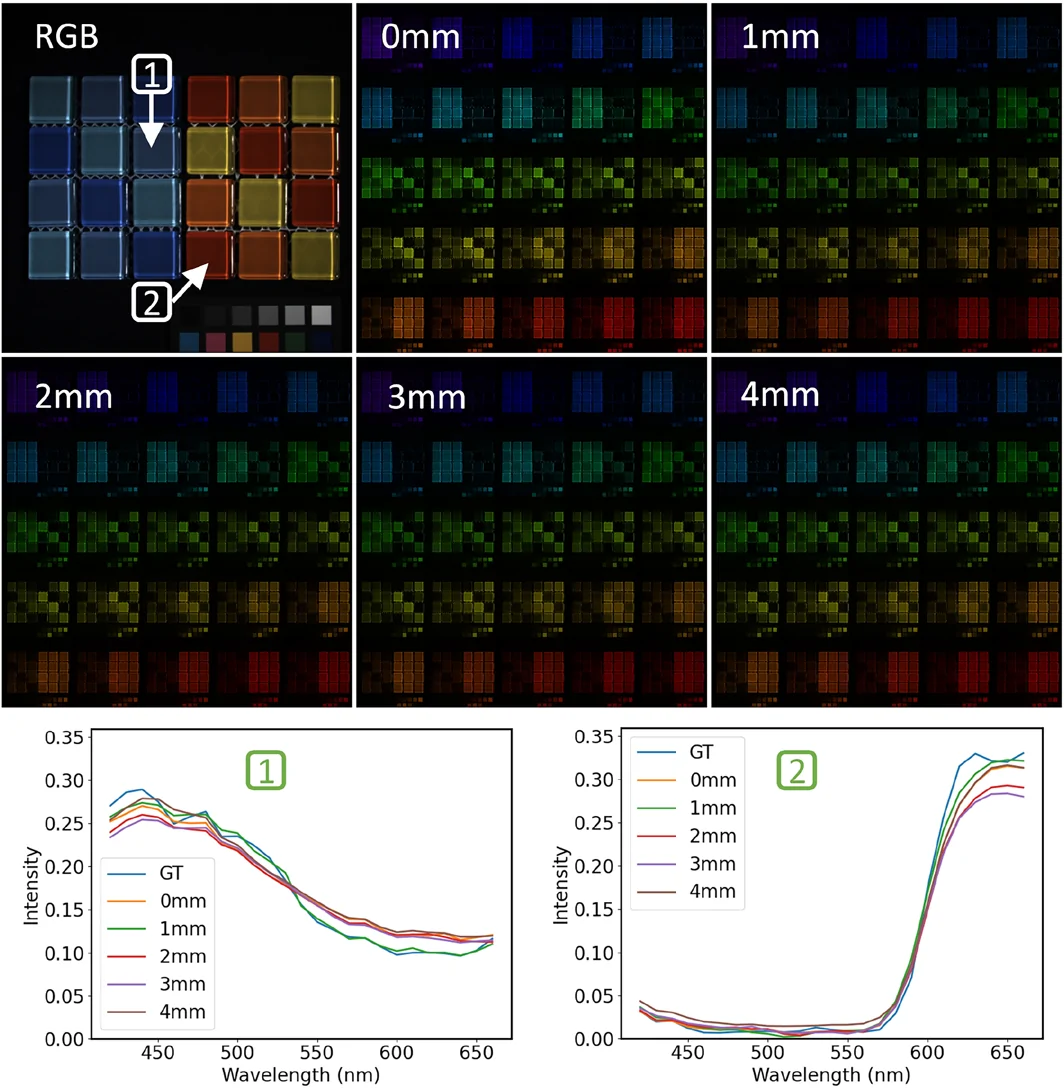
Comparison of spectral reconstruction under different lateral PSF shifts. The first and second rows show the ground‐truth RGB image and the hyperspectral image reconstruction results for various PSF lateral shifts. The third row compares the spectral profiles of selected representative pixels.

**TABLE 2:** Comparison of hyperspectral reconstruction under different lateral PSF shifts.

$\Delta_{\text{shift}}$ (mm)	SSIM	PSNR (dB)
0	0.94	30.26
1	0.96	32.54
2	0.92	29.89
3	0.90	29.25
4	0.89	28.97

### 2.6 Experimental Implementation

Through the end‐to‐end optimization, we obtained the structural design of the metalens. The detailed optimization procedure is provided in Supporting Information S1: Note S3. Each meta‐unit measured 400 nm × 400 nm, with an overall metalens diameter of 5 mm. We used two‐photon grayscale lithography to fabricate our metalens, controlling the phase precisely by tuning the height of each meta‐unit and discretizing the phase into 10 levels. The lithography process employed the IP‐DIP2 photoresist, which has a refractive index of 1.556 at the wavelength of 546.1 nm. Further details and analyses regarding the effect of height quantization and the dominant phase modulation mechanism of the meta‐units are provided in Supporting Information S1: Notes S4–S6.

Electron microscopy imaging of the fabricated metalens was performed and the results indicate a reasonable agreement between the designed and fabricated metalens structures, as shown in Figure 4. The metalens and an appropriately sized aperture were then assembled into a unit and installed on a Sony camera via a custom lens barrel, with the metalens positioned 36 mm in front of the sensor. The metalens orientation was manually adjusted to align the lateral shift direction of the PSFs with the width direction of the camera sensor, which has an image resolution of 24 megapixels and a pixel size of 5.94 μm. To ascertain the engineered dispersion characteristic of the metalens, a black‐and‐white checkerboard pattern was first captured using the hyperspectral camera prototype, and the output raw image is shown in Figure 4c. Noticeable lateral chromatic dispersion can be observed around the vertical edges of the checkerboard. A horizontal cross‐section is taken at the center of the image, and the corresponding RGB intensity profiles are plotted in Figure 4d. The results show that the RGB channels exhibit a clear chromatic dispersion shift along the lateral direction, which is consistent with our design expectations.

**FIGURE 4:**
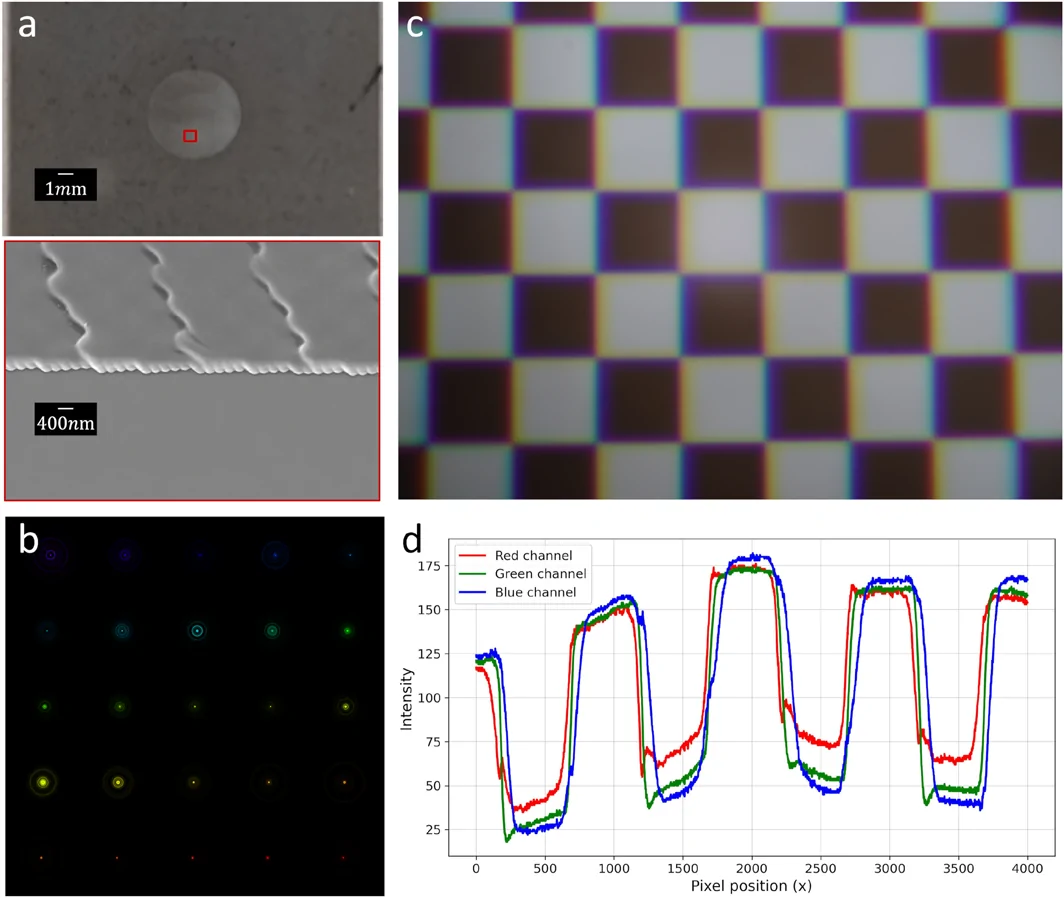
Fabricated metalens and its characterization. (a) Representative scanning electron microscopy images of a local region of the dispersion‐engineered metalens fabricated by two‐photon grayscale lithography. The metalens has a diameter of 5 mm, a meta‐unit period of 400 nm, and a focal length of 36 mm. (b) PSFs of the dispersion‐engineered metalens across 25 spectral bands from 420 to 660 nm, with a wavelength interval of 10 nm. (c) A raw checkerboard image captured by the hyperspectral camera. (d) RGB channel intensity profiles along the horizontal midline of the raw image.

Using the constructed hyperspectral camera prototype, we conducted imaging experiments in both indoor and outdoor scenarios. The results were compared with those from a commercial spectrometer (OHSP‐350) with a nominal spectral resolution of 0.2 nm to validate the effectiveness of the proposed method. Note that the employed commercial spectrometer acquires spectral information from only a single point, without any imaging functionality. As evidenced by Figure 5, the raw images captured by the sensor preserve the true colors and textures of the scenes, while exhibiting lateral dispersion at texture edges, which is consistent with the simulation results. Using the trained multi‐resolution spectral reconstruction network, the raw images are decoded into hyperspectral images, as shown in the second column. Based on the spectral response curves of the camera sensor, the hyperspectral images are synthesized to generate RGB images, as shown in the third column. The reconstructed RGB images ascertain high fidelity to the original images in visible color, partially validating the accuracy of the hyperspectral reconstruction. The fourth column provides direct comparisons between the reconstructed and ground‐truth spectral profiles. For a selection of representative image pixels of different colors, the spectra recovered by our method closely match those measured by the commercial spectrometer. These comparisons further demonstrate the reliability of the proposed approach in hyperspectral imaging and spectral reconstruction.

**FIGURE 5:**
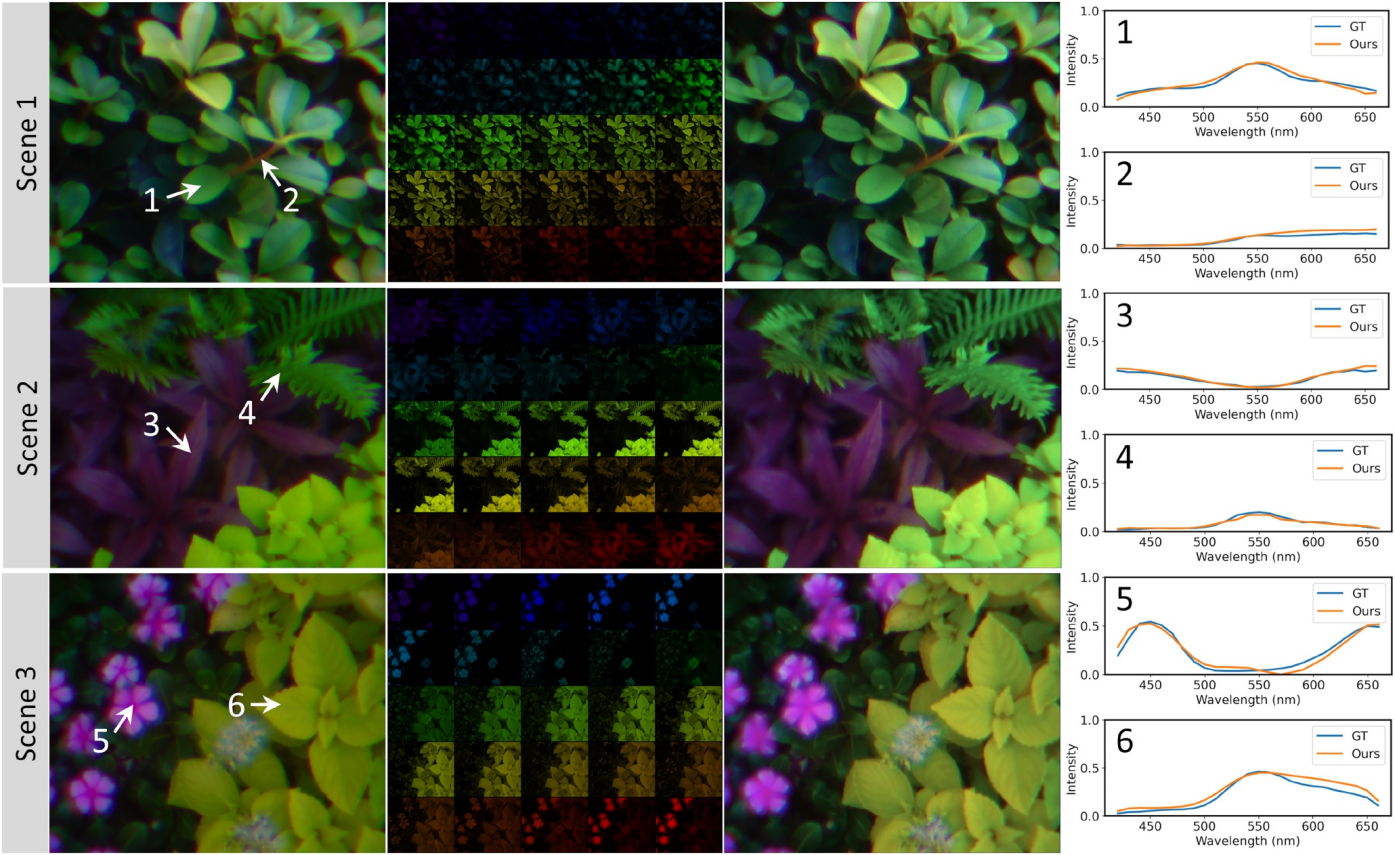
Hyperspectral reconstruction results on representative real‐world scenes. Here, three outdoor scenes of real plants are imaged. The first column shows the raw images captured by the camera sensor, the second column presents the reconstructed hyperspectral images, the third column displays the RGB images synthesized from the hyperspectral data, and the fourth column compares the spectral profiles from the proposed method versus those from a commercial spectrometer for selected representative pixels with different colors.

## 3 Discussion

In summary, we proposed a snapshot hyperspectral imaging method based on a single dispersion‐engineered metalens. The designed metalens exhibits wavelength‐dependent PSFs that undergo extra lateral shifts, thereby enabling more effective spectral encoding. To support this design, we devised an efficient strategy for computing the PSFs. Subsequently, we established a differentiable spectral forward‐encoding model and connected it with a downstream reconstruction network, forming an end‐to‐end differentiable hyperspectral imaging framework. By designing a hybrid loss function and training on available hyperspectral image datasets, we achieved a joint optimization of both the metalens structure and the spectral reconstruction network. Simulation results indicate that the introduction of moderate lateral dispersion can effectively enhance hyperspectral reconstruction performance while also demonstrating clear superiority of the proposed method over representative existing methods. We fabricated the learned metalens using two‐photon grayscale lithography and constructed a hyperspectral camera prototype. Experimental results show that the imaging system achieves accurate and robust spectral reconstruction in both indoor and outdoor scenarios, validating the feasibility and effectiveness in practical applications.

Although the proposed method demonstrates promising functional performance, there remains considerable room for improvement. For instance, the spectral dispersion information encoded by the dispersion‐engineered metalens is primarily concentrated at texture edges, which may lead to reduced reconstruction performance in sparsely textured scenes, even though the scenes in Figures 3 and 5 show that good performance can still be achieved in such cases. In the future, segmenting the imaging aperture for localized dispersion encoding could be explored to achieve superior spectral imaging performance across a wider range of scenes. However, the realization of this approach is contingent on the development of more advanced optical forward‐modeling. Nevertheless, this work provides valuable insights for the development of compact, lightweight, and high‐fidelity snapshot hyperspectral imaging systems with potential applications in remote sensing, biomedical imaging, industrial inspection, and consumer electronics. We also believe that the synergy between optical designs and computational frameworks will enable metasurfaces to extend their impact on hyperspectral imaging and beyond.

## 4 Experimental Section

The metalens was fabricated using a two‐photon grayscale lithography system (Nanoscribe Quantum X). After a set of process optimizations, the laser scanning linewidths were set at 200 nm in the horizontal direction and 100 nm in the vertical direction. Prior to fabrication, a fused silica substrate was prepared and cleaned with isopropanol. IP‐Dip2 photoresist was then dropped onto the center of the substrate, which was placed on the sample stage for focus alignment. The 3D nano‐lithography process was initiated using a 63 × objective lens, taking approximately 7.5 hours to complete. After nano‐lithography, the sample was developed in propylene glycol monomethyl ether acetate (PGMEA) solution for 20 minutes to fully remove unpolymerized photoresist from the substrate surface and finally cleaned in isopropyl alcohol (IPA) solution for 5 minutes.

## Author Contributions

**Peng Liu:** conceptualization, methodology, software, data curation, investigation, validation, formal analysis, writing – original draft, writing – review and editing. **Jiaru Chu:** supervision, resources, writing – review and editing. **Yuhang Chen:** writing – review and editing, project administration, resources, supervision, conceptualization, methodology, funding acquisition, visualization.

## Funding

The authors have nothing to report.

## Conflicts of Interest

The authors declare no conflicts of interest.

## Code Availability

The codes used for the end‐to‐end joint optimization are available from the corresponding author upon reasonable request.

## Supporting information


Supporting Information S1

## Data Availability

The data that support the findings of this study are available from the corresponding author upon reasonable request.

## References

[1] A. Bhargava , A. Sachdeva , K. Sharma , M. H. Alsharif , P. Uthansakul , and M. Uthansakul , “Hyperspectral Imaging and Its Applications: A Review,” Heliyon 10, no. 12 (2024): e33208, 10.1016/j.heliyon.2024.e33208. PMC11253060 39021975

[2] G. Tejasree and L. Agilandeeswari , “An Extensive Review of Hyperspectral Image Classification and Prediction: Techniques and Challenges,” Multimedia Tools and Applications 83, no. 34 (2024): 80941–81038, 10.1007/s11042-024-18562-9.

[3] V. Lodhi , D. Chakravarty , and P. Mitra , “Hyperspectral Imaging System: Development Aspects and Recent Trends,” Sensing and Imaging 20, no. 1 (2019): 35, 10.1007/s11220-019-0257-8.

[4] S. E. Qian , “Overview of Hyperspectral Imaging Remote Sensing From Satellites,” in Advances in Hyperspectral Image Processing Techniques, ed. C. Chang (Wiley‐IEEE Press, 2023), 41–66.

[5] A. Khan , A. D. Vibhute , S. Mali , and C. H. Patil , “A Systematic Review on Hyperspectral Imaging Technology With a Machine and Deep Learning Methodology for Agricultural Applications,” Ecological Informatics 69 (2022): 101678, 10.1016/j.ecoinf.2022.101678.

[6] J. Yoon , “Hyperspectral Imaging for Clinical Applications,” BioChip Journal 16 (2022): 1–12, 10.1007/s13206-021-00041-0.

[7] D.‐W. Sun , H. Pu , and J. Yu , “Applications of Hyperspectral Imaging Technology in the Food Industry,” Nature Reviews Electrical Engineering 1, no. 4 (2024): 251–263, 10.1038/s44287-024-00033-w.

[8] C. Ghisleri , M. A. C. Potenza , L. Ravagnan , A. Bellacicca , and P. Milani , “A Simple Scanning Spectrometer Based on a Stretchable Elastomeric Reflective Grating,” Applied Physics Letters 104, no. 6 (2014): 061910, 10.1063/1.4865427.

[9] G. R. Arce , D. J. Brady , L. Carin , H. Arguello , and D. S. Kittle , “Compressive Coded Aperture Spectral Imaging: An Introduction,” IEEE Signal Processing Magazine 31, no. 1 (2013): 105–115, 10.1109/msp.2013.2278763.

[10] X. Cao , H. Du , X. Tong , Q. Dai , and S. Lin , “A Prism‐Mask System for Multispectral Video Acquisition,” IEEE Transactions on Pattern Analysis and Machine Intelligence 33, no. 12 (2011): 2423–2435, 10.1109/tpami.2011.80. 21519097

[11] Y. Chen , J. Lv , W. Yue , et al., “A Snapshot Infrared Imaging Fourier Transform Spectrometer for Dynamic Target Detection,” Remote Sensing 14, no. 7 (2022): 1543, 10.3390/rs14071543.

[12] J. Xiong , X. Cai , K. Cui , et al., “Dynamic Brain Spectrum Acquired by a Real‐Time Ultraspectral Imaging Chip With Reconfigurable Metasurfaces,” Optica 9, no. 5 (2022): 461–468, 10.1364/optica.440013.

[13] K. Monakhova , K. Yanny , N. Aggarwal , and L. Waller , “Spectral DiffuserCam: Lensless Snapshot Hyperspectral Imaging With a Spectral Filter Array,” Optica 7, no. 10 (2020): 1298–1307, 10.1364/optica.397214.

[14] L. Li , L. Wang , W. Song , L. Zhang , Z. Xiong , and H. Huang , “Quantization‐Aware Deep Optics for Diffractive Snapshot Hyperspectral Imaging,” in Proceedings of the IEEE/CVF Conference on Computer Vision and Pattern Recognition (IEEE, 2022), 19780–19789, 10.1109/CVPR52688.2022.01916.

[15] D. S. Jeon , S.‐H. Baek , S. Yi , et al., “Compact Snapshot Hyperspectral Imaging With Diffracted Rotation,” ACM Transactions on Graphics 38, no. 4 (2019): 117, 10.1145/3306346.3322946.

[16] Z. Shi , X. Dun , H. Wei , et al., “Learned Multi‐Aperture Color‐Coded Optics for Snapshot Hyperspectral Imaging,” ACM Transactions on Graphics 43, no. 6 (2024): 208–211, 10.1145/3687976.

[17] K. Cui , S. Rao , S. Xu , et al., “Spectral Convolutional Neural Network Chip for In‐Sensor Edge Computing of Incoherent Natural Light,” Nature Communications 16, no. 1 (2025): 81, 10.1038/s41467-024-55558-3. PMC11696300 39747892

[18] Y. Zhang , X. Jiang , G. Qu , et al., “On‐Chip Integration of Achromatic Metalens Arrays,” Nature Communications 16, no. 1 (2025): 7485, 10.1038/s41467-025-62539-7. PMC12343996 40796744

[19] A. H. Dorrah and F. Capasso , “Tunable Structured Light With Flat Optics,” Science 376, no. 6591 (2022): eabi6860, 10.1126/science.abi6860. 35446661

[20] P. Liu , F. Xu , J. Chu , and Y. Chen , “Multichannel Fusion Structured Light 3D Imaging Based on Metasurfaces,” Laser & Photonics Reviews 19, no. 13 (2025): 2402297, 10.1002/lpor.202402297.

[21] F. Xu , W. Chen , M. Li , P. Liu , and Y. Chen , “Broadband Achromatic and Wide Field‐of‐View Single‐Layer Metalenses in the Mid‐Infrared,” Optics Express 31, no. 22 (2023): 36439–36450, 10.1364/oe.504892. 38017797

[22] A. Zaidi , N. A. Rubin , M. L. Meretska , et al., “Metasurface‐Enabled Single‐Shot and Complete Mueller Matrix Imaging,” Nature Photonics 18, no. 7 (2024): 704–712, 10.1038/s41566-024-01426-x.

[23] Z. Fan , C. Qian , Y. Jia , et al., “Holographic Multiplexing Metasurface With Twisted Diffractive Neural Network,” Nature Communications 15, no. 1 (2024): 9416, 10.1038/s41467-024-53749-6. PMC11528057 39482288

[24] L. Zhang , C. Zhou , B. Liu , et al., “Real‐Time Machine Learning‐Enhanced Hyperspectro‐Polarimetric Imaging via an Encoding Metasurface,” Science Advances 10, no. 36 (2024): eadp5192, 10.1126/sciadv.adp5192. PMC11373597 39231222

[25] Y. Ning , J. Zhu , P. Ji , et al., “Ultra‐Compact on‐Chip Snapshot Chiral Spectral Imaging With Dispersion‐Engineered Chiral Metalens,” npj Nanophotonics 2, no. 1 (2025): 23, 10.1038/s44310-025-00066-5.

[26] C.‐H. Lin , S.‐H. Huang , T.‐H. Lin , and P. C. Wu , “Metasurface‐Empowered Snapshot Hyperspectral Imaging With Convex/Deep (CODE) Small‐Data Learning Theory,” Nature Communications 14, no. 1 (2023): 6979, 10.1038/s41467-023-42381-5. PMC10620425 37914700

[27] Y. He , M. K. Chen , M. Huang , et al., “Dispersive Meta‐Lens Thermometry for High‐Temperature Measurements,” Nature Communications 16, no. 1 (2025): 10090, 10.1038/s41467-025-65171-7. PMC12627435 41253803

[28] J. E. Fröch , S. Colburn , A. Zhan , et al., “Dual Band Computational Infrared Spectroscopy via Large Aperture Meta‐Optics,” ACS Photonics 10 (2023): 986–992, 10.1021/acsphotonics.2c01017.

[29] M. Gopakumar , G.‐Y. Lee , S. Choi , et al., “Full‐Colour 3D Holographic Augmented‐Reality Displays With Metasurface Waveguides,” Nature 629, no. 8013 (2024): 791–797, 10.1038/s41586-024-07386-0. PMC11111399 38720077

[30] E. Choi , G. Kim , J. Yun , Y. Jeon , J. Rho , and S.‐H. Baek , “360° Structured Light With Learned Metasurfaces,” Nature Photonics 18, no. 8 (2024): 848–855, 10.1038/s41566-024-01450-x.

[31] C.‐F. Pan , H. Wang , H. Wang , et al., “3D‐Printed Multilayer Structures for High‐Numerical Aperture Achromatic Metalenses,” Science Advances 9, no. 51 (2023): eadj9262, 10.1126/sciadv.adj9262. PMC10732525 38117894

[32] L. Zhang , H. Wang , Q. Jiang , L. Han , X. Zhang , and S. Zhuang , “Dual‐Wavelength Multiplexed Metasurface Holography Based on Two‐Photon Polymerization Lithography,” Nanophotonics 14, no. 5 (2025): 581–588, 10.1515/nanoph-2024-0705. PMC11953720 40161537

[33] J. Yang , K. Cui , X. Cai , et al., “Ultraspectral Imaging Based on Metasurfaces With Freeform Shaped Meta‐Atoms,” Laser & Photonics Reviews 16, no. 7 (2022): 2100663, 10.1002/lpor.202100663.

[34] M. Makarenko , A. Burguete‐Lopez , Q. Wang , et al., “Real‐Time Hyperspectral Imaging in Hardware via Trained Metasurface Encoders,” in Proceedings of the IEEE/CVF Conference on Computer Vision and Pattern Recognition (IEEE, 2022), 12692–12702, 10.1109/CVPR52688.2022.01236.

[35] N. Xie , V. Saragadam , J. E. Fröch , K. F. Böhringer , and A. Majumdar , “Snap‐Shot Hyperspectral Imaging Enabled by Metasurface,” Nano Letters 26, no. 4 (2026): 1246–1253, 10.1021/acs.nanolett.5c04868. 41586810

[36] S.‐J. Cho , S.‐W. Ji , J.‐P. Hong , S.‐W. Jung , and S.‐J. Ko , “Rethinking Coarse‐to‐Fine Approach in Single Image Deblurring,” Proceedings of the IEEE/CVF International Conference on Computer Vision (IEEE, 2021), 4641–4650, 10.1109/ICCV48922.2021.00460.

[37] K. Simonyan and A. Zisserman , “Very Deep Convolutional Networks for Large‐Scale Image Recognition,” preprint, arXiv, 2014, 10.48550/arXiv.1409.1556.

[38] B. Arad and O. Ben‐Shahar , “Sparse Recovery of Hyperspectral Signal From Natural RGB Images,” in European Conference on Computer Vision (Springer, 2016), 19–34, 10.1007/978-3-319-46478-7_2.

[39] F. Yasuma , T. Mitsunaga , D. Iso , and S. K. Nayar , “Generalized Assorted Pixel Camera: Postcapture Control of Resolution, Dynamic Range, and Spectrum,” IEEE Transactions on Image Processing 19, no. 9 (2010): 2241–2253, 10.1109/tip.2010.2046811. 20350852

[40] Y. Li , Q. Fu , and W. Heidrich , “Multispectral Illumination Estimation Using Deep Unrolling Network,” in Proceedings of the IEEE/CVF International Conference on Computer Vision (IEEE, 2021), 2672–2681, 10.1109/ICCV48922.2021.00267.

[41] A. Chakrabarti , and T. Zickler , “Statistics of real‐world Hyperspectral Images,” in Proceedings of the IEEE/CVF Conference on Computer Vision and Pattern Recognition (IEEE, 2011), 193–200, 10.1109/CVPR.2011.5995660.

