# Supplementary material for: Compact Snapshot Hyperspectral Imaging With Neural Dispersion‐Engineered Metalens
Source: Nanophotonics. 2026 Aug 7;15(15):e70245. doi: 10.1002/nap2.70245 (PMC13642530; doi:10.1002/nap2.70245)
Supplement: Supplementary file 1 [file NAP2-15-e70245-s001.docx]

**Supplementary Information**

**Compact Snapshot Hyperspectral Imaging with Neural Dispersion-Engineered Metalens**

Peng Liu, Jiaru Chu, and Yuhang Chen^*^

Department of Precision Machinery and Precision Instrumentation, University of Science and Technology of China, Hefei 230027, China

^*^Corresponding author. Email: [chenyh@ustc.edu.cn](mailto:chenyh@ustc.edu.cn)

**Supplementary** **Note 1: Simulation method for the dispersion-engineered metalens**

Here, we proceed from Equation (4) in the main text to carry out further mathematical derivations.

$PSF(x,y,\lambda)=\left| \frac{e^{iks}}{i\lambda s}\iint U^{'}(u,v)e^{i\frac{k}{2s}(\left( x-u \right)^{2}+\left( y-v \right)^{2}))}dudv \right|^{2}$	(S1)

By substituting into the equation and omitting constant terms, we obtain,

$PSF(x,y,\lambda)=\left| \frac{1}{\lambda s}\iint p(u,v)e^{i\varphi_{total}}e^{\frac{i\pi}{\lambda}\left( \frac{1}{z}+\frac{1}{s} \right)\left( u^{2}+v^{2} \right)}e^{-i\frac{2\pi}{\lambda s}\left( ux+vy \right)}dudv \right|^{2}$ (S2)

Here, $\varphi_{total}=\varphi_{focus}+\varphi_{shift}$, and $\varphi_{shift}=\frac{2\pi}{\lambda s}\Delta_{shift}u$.

Let $H\left( \frac{2\pi}{\lambda s}u, \frac{2\pi}{\lambda s}v \right)=p(u,v)e^{i\varphi_{focus}}e^{\frac{i\pi}{\lambda}\left( \frac{1}{z}+\frac{1}{s} \right)\left( u^{2}+v^{2} \right)}$, the above equation becomes,

$PSF(x,y,\lambda)=\left| \frac{1}{\lambda s}\iint H\left( \frac{u}{\lambda s}, \frac{v}{\lambda s} \right)e^{i\frac{2\pi}{\lambda s}\Delta_{shift}u}e^{-i\frac{2\pi}{\lambda s}\left( ux+vy \right)}dudv \right|^{2}$	(S3)

Let $F\left( x,y,\lambda\right)\mathcal{=F}\left[ H\left( \frac{u}{\lambda s}, \frac{v}{\lambda s} \right) \right]$, then we have $PSF(x,y,\lambda)=\left| F\left( x-\Delta_{shift},y,\lambda\right) \right|^{2}$.

Accordingly, the computation of $PSF(x,y,\lambda)$ can be decomposed into two steps: first, applying the Fourier transform to $H\left( \frac{u}{\lambda s}, \frac{v}{\lambda s} \right)$, and second, introducing a lateral displacement $\Delta_{shift}$ in the sensor plane. Given that the number of meta-units in the metalens to be optimized reaches approximately 120 million, directly computing their Fourier transform with conventional methods would demand prohibitive computational resources. Moreover, simultaneously optimizing such a massive number of parameters is highly challenging. To mitigate the computational burden, we introduce the assumption of rotational symmetry in the metasurface structure, thereby transforming the Fourier transform of $H\left( \frac{2\pi}{\lambda s}u, \frac{2\pi}{\lambda s}v \right)$ from Cartesian to polar coordinates ^[1]^,

$F\left( \rho,\phi,\lambda\right)=\frac{2\pi}{\lambda s}\int_{0}^{\infty} rH(r,\lambda)dr\int_{0}^{2\pi} exp\left[ -j2\pi r\rho cos(\theta-\phi) \right]d\theta$	(S4)

Applying the Bessel identity $J_{0}\left( a \right)=\frac{1}{2\pi}\int_{0}^{2\pi} exp\left[ -jacos(\theta-\phi) \right]d\theta$, we can derive,

$F\left( \rho,\phi,\lambda\right)=\frac{2\pi}{\lambda s}\int_{0}^{\infty} rH(r,\lambda)J_{0}(2\pi r\rho)dr$	(S5)

By discretizing this integral expression, we obtain,

$$F\left( \rho,\phi,\lambda\right)=\frac{2\pi}{\lambda s}\int_{0}^{R} rH\left( r,\lambda\right)J_{0}\left( 2\pi r\rho\right)dr$$

$=\frac{2\pi}{\lambda s}\sum_{l=0}^{L-1} \int_{l\Delta}^{\left( l+1 \right)\Delta} rH\left( r,\lambda\right)J_{0}\left( 2\pi r\rho\right)dr$

$$\approx\frac{2\pi}{\lambda s}\sum_{l=0}^{L-1} H\left( l\Delta,\lambda\right)\int_{l\Delta}^{\left( l+1 \right)\Delta} rJ_{0}\left( 2\pi r\rho\right)dr$$

$$=\frac{2\pi}{\lambda s}\sum_{l=0}^{L-1} H\left( l\Delta,\lambda\right)\int_{0}^{\left( l+1 \right)\Delta} rJ_{0}\left( 2\pi r\rho\right)dr- \int_{0}^{L\Delta} rJ_{0}\left( 2\pi r\rho\right)dr]$$

$=\left\{ \begin{aligned} \frac{2\pi}{\lambda s}\sum_{l=0}^{L-1} H\left( l\Delta,\lambda\right)\left[ \frac{{((l+1)\Delta)}^{2}}{2}-\frac{({l\Delta)}^{2}}{2} \right] if \rho=0 \\ \frac{2\pi}{\lambda s}\sum_{l=0}^{L-1} H\left( l\Delta,\lambda\right)\left[ \frac{\left( l+1 \right)\Delta}{2\pi\rho}J_{1}\left( 2\pi\left( l+1 \right)\Delta\rho\right)-\frac{l\Delta}{2\pi\rho}J_{1}(2\pi l\Delta\rho) \right] otherwise \end{aligned} \right\}$ (S6)

In Equation (S6), we discretize the aperture radius $R$ of the metalens as $l\Delta$, and $J_{1}$ denotes the first-order Bessel function. Subsequently, the PSF displacements at other wavelengths are calculated based on the lateral displacement measured at the central wavelength. Let the central wavelength be $\lambda_{1}$ with a corresponding PSF displacement $\Delta_{1}$ and refractive index $n_{1}$, and the target wavelength be $\lambda_{2}$ with the corresponding lateral PSF displacement $\Delta_{2}$ and refractive index $n_{2}$. Based on $h=[\lambda/{2\pi\left( n-1 \right)]p}$ and $p={(2\pi}/{\lambda)x\Delta/f}$, we can derive,

$\frac{dh}{dx}=\frac{dh}{dp}\frac{dp}{dx}$	(S7)

$\frac{dh}{dp_{1}}\frac{dp_{1}}{dx}=\frac{dh}{dp_{2}}\frac{dp_{2}}{dx}$	(S8)

By substituting the above equation, we obtain,

$\Delta_{2}=\frac{n_{2}-1}{n_{1}-1}\Delta_{1}$	(S9)

Thus, the PSF displacements at other wavelengths can be directly obtained from the lateral displacement of the PSF at the assigned center wavelength.

Based on the above analysis, the spectral resolution introduced by the lateral dispersion alone can be further estimated. Assuming that two wavelengths are considered resolvable when the distance between their corresponding PSF centers exceeds one camera pixel (5.94$\mu m$), the spectral resolution introduced by lateral dispersion can be calculated using Equation (S9) together with the refractive index of polymerized IP-Dip2 photoresist shown in Figure S3. The calculated spectral resolution is typically 14.65 nm at 420 nm. It should be noted that the practically achievable spectral resolution can be much higher with the assistance of the PSF radial dispersion and the spectral reconstruction network.

**Supplementary Note 2: Details of the multi-resolution hyperspectral reconstruction network architecture**

As described in Section 2.3 in the main text, we designed a multi-resolution parallel neural network to reconstruct the hyperspectral information, which is then used to synthesize RGB images. The encoded raw RGB images are fed into the network in parallel at three different scales, with inter-layer connections implemented via upsampling and downsampling to facilitate information exchange. The overall network architecture is shown in Figure S1, while Table S1 provides detailed specifications of the network.

**Figure S1. The multi-resolution network for hyperspectral reconstruction.**

**Table S1. Details of the network architecture.**

| Input | Layer Type | Output |
| --- | --- | --- |
| Sensor_image1 | Conv_3_1b_k5_s1_LRelu  ResConv_1b_1b_k3_s1_LRelu | X_00 |
| X_00 | Conv_1b_2b_k3_s2_LRelu | X_00_down |
| X_00 | Conv_1b_1b_k3_s1_LRelu  ResConv_1b_1b_k3_s1_LRelu | X_01 |
| Sensor_image2  _00_down | Conv_2b+3_2b_k3_s1_LRelu  ResConv_2b_2b_k3_s1_LRelu | X_11 |
| X_11 | Convtransp_2b_1b_k3_s2_LRelu | X_11_up |
| X_11 | Conv_2b_4b_k3_s2_LRelu | X_11_down |
| X_01  X_11_up1 | Conv_2b_1b_k3_s1_LRelu  ResConv_1b_1b_k3_s1_LRelu | X_02 |
| X_02 | Conv_1b_2b_k3_s2_LRelu | X_02_down1 |
| X_01 | Conv_1b_2b_k3_s2_LRelu | X_01_down1 |
| X_01 | Conv_1b_4b_k3_s4_LRelu | X_01_down2 |
| X_01_down1  X_11 | Conv_4b_2b_k3_s1_LRelu  ResConv_2b_2b_k3_s1_LRelu | X_12 |
| X_12 | Convtransp_2b_1b_k3_s2_LRelu | X_12_up1 |
| Sensor_image3  X_01_down2  X_11_down1 | Conv_8b+3_4b_k3_s1_LRelu  ResConv_4b_4b_k3_s1_LRelu | X_22 |
| X_22 | Conv_4b_2b_k3_s2_LRelu | X_22_up1 |
| X_22 | Conv_4b_1b_k3_s4_LRelu | X_22_up2 |
| X_02  X_12_up1  X_22_up2 | Conv_3b_1b_k3_s1_LRelu  ResConv_1b_1b_k3_s1_LRelu | X_03 |
| X_02_down1  X_12  X_22 | Conv_6b_2b_k3_s1_LRelu  ResConv_2b_2b_k3_s1_LRelu | X_13 |
| X_13 | Convtransp_2b_1b_k3_s2_LRelu | X_13_up1 |
| X_03  X_13_up1 | Conv_3b_1b_k3_s1_LRelu  ResConv_1b_1b_k3_s1_LRelu | X_04 |
| X_04 | Conv_b_25_k3_s1_LRelu | Spectral1 |
| X_13 | Conv_2b_25_k3_s1_LRelu | Spectral2 |
| X_22 | Conv_3b_25_k3_s1_LRelu | Spectral3 |

Here, Conv_n1(b)_n2(b)_(k)n3_s(n4)_LRelu represents a convolutional block, where the number of input channels is n1(b), the number of output channels is n2(b), the kernel size is n3×n3, the stride is n4, and the activation function is LeakyReLU. Similarly, ResConv_n1(b)_n2(b)_(k)n3_s(n4)_LRelu denotes the corresponding residual convolutional block, while Convtransp_n1(b)_n2(b)_(k)n3_s(n4)_LRelu denotes the corresponding transposed convolutional block.

**Supplementary Note 3: Optimization workflow of the neural dispersion-engineered metalens**

As stated in Equation (2) in the main text, the phase of the metalens consists of two components. The first component is the base focus phase, which provides the fundamental focus functionality and realizes part of the spectral encoding. This phase component is optimized through the end-to-end framework. The second component is the linear gradient phase, which introduces wavelength-dependent dispersion shifts to further enrich the spectral encoding. Here, we present the detailed optimization and computation workflow for the phase and the metalens structure.

First, we design a quadratic phase profile centered at 546.1 nm,

${phase\_focus}_{raw}=-\frac{\pi}{\lambda f}(x^{2}+y^{2})$	(S10)

This phase is directly superimposed with the linear gradient phase,

${phase\_total}_{raw}={phase\_focus}_{raw}+phase\_gradient$	(S11)

Subsequently, the focusing phase term and the spectral reconstruction algorithm are jointly optimized through the end-to-end framework to obtain the optimized phase,

${phase\_total}_{op}={phase\_focus}_{op}+phase\_gradient$	(S12)

Then, the structure of the metalens can be computed from the optimized phase,

${heightmap}_{op}={phase\_total}_{op}\times\frac{\lambda}{2\pi(n-1)}$	(S13)

It is worth noting that once the lens structure becomes essentially stable during the optimization process, the structure is fixed. At that stage, the joint optimization is switched to fine-tuning only the spectral reconstruction algorithm. Figure S2 shows one typical optimized heightmap of the metalens.

**Figure S2. Typical height map of the optimized metalens with the lateral PSF shift of 1 mm at the central wavelength.** The metalens diameter is 5 mm.

**Supplementary Note 4: Refractive index of IP-Dip2**

The refractive index data of the polymerized IP‑Dip2 photoresist provided by the manufacturer are listed in Table S2.

**Table S2. Refractive index of IP-Dip2 photoresist.**

| Wavelength (nm) | 405 | 479.3 | 546.1 | 589.3 | 643.8 | 780 | 950 |
| --- | --- | --- | --- | --- | --- | --- | --- |
| Refractive index | 1.579 | 1.564 | 1.556 | 1.552 | 1.548 | 1.543 | 1.539 |

The wavelength dependence of the refractive index shown in Figure S3 was obtained by fitting using the Cauchy equation, which was used for the metalens design.

**Figure S3. Wavelength dependence of the refractive index of polymerized IP‑Dip2 photoresist.**

**Supplementary Note 5: Effect of height quantization on the spectral reconstruction**

To investigate the impact of 10-level phase quantization on spectral reconstruction, a comparison is conducted between the reconstruction results before and after quantization. Considering that the axial slicing thickness of two-photon grayscale lithography is 100 nm, the height is quantized using a step size of 100 nm. Figure S4 compares the spectral reconstruction results under these two conditions, yielding a SSIM of 0.998. The results indicate that height quantization has a negligible effect on the spectral reconstruction.

**Figure S4. Comparison of spectral reconstruction results before and after quantization.** (a) Spectral reconstruction results before quantization. (b) Spectral reconstruction results after quantization. (c) Comparison of the reconstructed spectral profiles at a single randomly selected pixel location.

**Supplementary Note 6: Phase modulation of meta-units**

To demonstrate that the phase modulation of the meta-units employed in this work primarily originates from the propagation phase, which is dependent on the height, finite-difference time-domain (FDTD) simulations are performed with a height sweep of the meta-unit. The height is varied from 0 μm to 1 μm with a step of 100 nm, and the corresponding modulated phase is extracted. The simulation results are shown in Figure S5. It can be observed that the phase modulation of the meta-units is mainly governed by the propagation phase associated with the height.

**Figure S5. FDTD simulation results of meta-unit phase modulation.**

**References**

[1] H. Ikoma, C. M. Nguyen, C. A. Metzler et al., "Depth from defocus with learned optics for imaging and occlusion-aware depth estimation," in *2021 IEEE International Conference on Computational Photography*, pp. 1-12.
